# Up-Regulated Expression and Aberrant DNA Methylation of *LEP* and *SH3PXD2A* in Pre-Eclampsia

**DOI:** 10.1371/journal.pone.0059753

**Published:** 2013-03-27

**Authors:** Yuqian Xiang, Yan Cheng, Xiaotian Li, Qiaoli Li, Jiawei Xu, Junyu Zhang, Yun Liu, Qinghe Xing, Lei Wang, Lin He, Xinzhi Zhao

**Affiliations:** 1 Children’s Hospital and Institutes of Biomedical Sciences, Fudan University, Shanghai, China; 2 Obstetrics & Gynecology Hospital of Fudan University, Shanghai, China; 3 Bio-X Institutes, Key Laboratory for the Genetics of Developmental and Neuropsychiatric Disorders (Ministry of Education), Shanghai Jiao Tong University, Shanghai, China; VU University Medical Center, The Netherlands

## Abstract

The primary mechanism underlying pre-eclampsia (PE) remains one of the most burning problems in the obstetrics and gynecology. In this study, we performed an expression profiling screen and detected 1312 genes that were differentially expressed (*p*<0.05 and fold change >1.5) in PE placentas, including *LEP* and *SH3PXD2A*. After validating the microarray results, we conducted the quantitative methylation analysis of *LEP* and *SH3PXD2A* in preeclamptic (n = 16) versus normal placentas (n = 16). Our results showed that many CpG sites close to the transcriptional start site (TSS) of *LEP* gene were hypomethylated in placentas from pregnancies with PE compared with those of in controls, including the TSS position (*p* = 0.001), the binding sites of Sp1 (*p* = 1.57×10^−4^), LP1 (*p* = 0.023) and CEBPα (*p* = 0.031). Luciferase reporter analysis confirmed the aberrant methylation of *LEP* promoter and CEBPα co-transfection had a role in the regulation of gene expression. Our results indicated the aberrant *LEP* promoter methylation was involved in the development of PE. We did not find a significant methylation differences between groups in the promoter region of *SH3PXD2A*, however, a CGI region in the gene body (CGI34) presented a higher methylation in preeclamptic placentas (*p* = 1.57×10^−4^), which might promote the efficiency of gene transcription. We speculated that SH3PXD2A may take part in the pathogenesis of PE through its role in the regulation of trophoblast cell invasion in the period of placenta formation.

## Introduction

Pre-eclampsia (PE; MIM 189800), a devastating pregnancy-associated disorder, is characterized by *de-novo* hypertension and proteinuria at or after 20 weeks’ gestation. The disease has a prevalence of 5–8% in nulliparous pregnancies and has been a leading cause of perinatal materno-foetal morbidity and mortality worldwide [1]–[3]. The World Health Organization (WHO) Nations has proposed the goals of PE treatment by reducing maternal mortality by 75% between 1990 and 2015 [4]. A great deal of researches have been conducted to explore the precise etiology of PE over the past decades. Some progress has been made toward the emerging consensus that PE is a heterogenic multisystem disorder and that a variety of theories such as endothelial dysfunction [5], [6], maternal-fetal (paternal) immune maladaptation [7], [8] and dysregulated inflammatory factors [9] have been suggestive of being the contributors to PE occurrence. To date, no definitive therapeutic interventions have been identified; and the only effective cure to treat PE is delivery of the placenta [3], which would lead to low birth-weight infant that brings about long-term adverse health effects such as cardiovascular disease [10]. Hence, it is of great significance to understand the underlying pathogenesis of the disease.

DNA methylation, the most extensively studied epigenetic modification, has been indicated to be involved in the development of human diseases [11]–[15]. Consequently, it is essential to understand the roles of DNA methylation in disease processes. Numerous studies have indicated high homocysteine in the plasma of pregnancies with PE [16]–[19], while multivitamin supplements containing folic acid and vitamin B_12_ are correlated with reduced risk of PE [17], [19], which indicate the potential role of DNA methylation in the pathophysiology of PE. Furthermore, to acquire a deep insight into PE at the molecular level, the association of DNA methylation with PE has been intensively studied both in the gene-specific pattern [20]–[22] and in the genome-wide level [17], [23]. In addition to the well-defined *SERPINA3* [22], *APC* [21] and *TIMP3* [23], [24], a series of genes with differential methylation related to collagen metabolism, angiogenesis and blood vessel development have also been identified to be involved in PE with aberrant methylation patterns [17], [25]. Given all that, DNA methylation appears to be a significant factor in the development of PE.

The causal relationship between promoter DNA methylation aberrations and gene expression differences has been well established. Promoter hypermethylation has been shown to associate with transcriptional repression and hence decreases expression and functional gene dosage; while demethylation in the promoter region will increase the transcription. Given the association of DNA methylation with PE mentioned above, we want to make an investigation of genes and functional networks with regard to epigenetics. So far, comparative gene expression profiling analyses of normal and pathological placentas have identified subsets of genes with altered expression [26]; however, few studies have been performed to explore the mechanisms that underlie the differential gene expression. Consequently, it is valuable to estimate the relative contribution of changes in DNA methylation levels to gene expression differences.

In the current study, we have performed a global gene expression profiling via oligonucleotide microarrays of placental tissue from preeclamptic pregnancies and uncomplicated pregnancies to explore genes with differential expression. Among the differentially expressed genes in our study, we picked up *LEP*, the gene with biggest expression difference, and *SH3PXD2A*, the gene with the most significant *p* value, for further analysis to explore the relevance of DNA methylation in the development of PE as increasing studies suggested that aberrant DNA methylation was considered as a pathogenic factor in the onset of PE [20], [27], [28].

## Materials and Methods

### Ethics Statements

The study protocols were approved by the Ethics Review Committee of Fudan University and conducted according to the Declaration of Helsinki Principles. All participants in this manuscript have given written informed consent (as outlined in the PLoS consent form) to publish their details.

### Patients and Samples

Placental tissues were obtained from pregnancies with PE (n = 23) and from uncomplicated pregnancies (n = 22) with singleton. All participants in the present study are Han Chinese in origin. Usually, diagnostics criteria used for PE patients were as follows: systolic pressure >140 mmHg, diastolic pressure >90 mmHg, and proteinuria >0.3 g in a 24 hours collection. The controls comprised the pregnancies undergoing caesarean section without suffering from other diseases. Clinical characteristics of all participants are shown in Table 1. For the microarray experiment, samples from 5 women with PE and 7 uncomplicated pregnancies were collected. For quantitative real-time PCR (qRT-PCR) validation, additional 7 preeclamptic pregnancies and 6 normotensive pregnancies were included. For DNA methylation analysis, 16 clinical samples with PE and 16 control samples including samples used in microarray analysis were used to perform DNA methylation analysis. For linear correlation analysis, 12 placentas from normotensive pregnancies (5 placentas used in qRT-PCR and 7 placentas used in microarray analysis) were included. Materials of some placentas used in this study have been published in our previous study [29]. All clinical placentas from normal and pathological pregnancies were collected immediately after the caesarean section. Two ∼1 cm^3^ fragments were dissected from the placenta, after removal of maternal blood by vigorous washing in phosphate buffered saline (PBS). The tissues were maintained in centrifuge tubes and RNAlater (Ambion Inc., Austin, TX), and then frozen at −80°C.

**Table 1:** Clinical characteristics of the study population.

Characteristic	PE (n = 23)^a^	Control (n = 22)	*p*-value^b^
Maternal age (years)	30.95±6.48	28.5±3.73	0.387
Gestational age (weeks)	35.34±3.07	39.38±1.19	1.02×10^−5^
Pregnancy BMI (kg/m^2^)	30.72±3.49	31.45±3.76	0.616
Systolic BP (mmHg)	154.18±17.85	108.56±12.84	8.99×10^−11^
Diastolic BP (mmHg)	106.27±15.07	68.69±8.93	8.99×10^−11^
Proteinuria (g/24 h)	4.06±4.63	0	1.55×10^−10^
Infant birthweight (g)	2281.5±969.90	3406±293.83	2.94×10^−4^
Plcenta weight (g)	402.6±143.29	692.14±74.38	2.35×10^−5^

All results are presented as mean ± SD.

a Diagnostics criteria used for PE patients were as follows: systolic pressure >140 mmHg, diastolic pressure >90 mmHg, and proteinuria >0.3 g in a 24 hour collection.

b Obtained using the Mann-Whitney *U*-test on SPSS version 16.0 (SPSS Inc., Chicago, IL, USA).

### RNA Preparation

Total RNAs were extracted from placentas using the mirVana™ miRNA Isolation Kit (Ambion) according to manufacturer’s instruction, including a DNase I digestion step. The quality of RNA was determined by Nanodrop ND-1000 (NanoDrop Technologies, Wilmingon, USA) and electrophoresis through denaturing gels.

### Microarray Analysis

Microarray experiments were performed using the Roche Nimblegen Gene Expression 12×135 K Arrays. A total of 5 placentas from pregnancies with PE and 7 from normal subjects were included as discovery round samples in the hybridizations. Raw data were extracted as pair files by NimbleScan software (version 2.5), and Robust multi-array average (RMA) method was used to offer quantile normalization and background correction. The primary microarray data have been submitted to Gene Expression Omnibus with accession number GSE43942. To identify the differentially expressed genes, student’s *t*-test analysis was performed. The threshold we used to screen up or down-regulated genes is fold change > = 1.5 with a *p* value cut-off of <0.05. Gene Ontology (GO) and annotation analysis was conducted using DAVID Tools [30] for function analysis of the screened differentially expressed genes.

### cDNA Preparation and Quantitative Real-time PCR

Reverse transcription was conducted with 1 µg RNA using M-MLV Reverse Transcriptase (Promega, Madison, WI, USA). qRT-PCR was performed to determine the mRNA expression of *LEP* and *SH3PXD2A* with FastStart Universal SYBR Green master (ROX) reagent (Roche Diagnostics, Basel, Switzerland) in 7900HT Fast Real-Time PCR System (Applied Biosystems, Foster City, CA). An endogenous control gene, *GAPDH* was used as an internal control to normalize cDNA loadings among samples. Optimal qRT-PCR assay for *LEP, SH3PXD2A* and *GAPDH* (PCR primers were listed in Table S1) were designed on PrimerBank (http://pga.mgh.harvard.edu/primerbank). All qRT-PCR reactions were performed in replicates in a final volume of 10 µl containing primers, FastStart Universal SYBR Green master (ROX) reagent (Roche) and cDNA sample. Relative expression of selected genes in samples was quantified by normalized against *GAPDH* according to the 2^−ΔΔCt^ method.

### DNA Preparation, Bisulphite Conversion and Quantitative Methylation Analysis

Genomic DNA was isolated from placentas using the QIAamp DNA Mini Kit (QIAGEN, Hilden, Germany). DNA was bisulphite-converted with the EZ DNA Methylation Kit (Zymo Research, Orange, CA, USA) according to the manufacturer’s protocol. Quantitative methylation of *LEP* and *SH3PXD2A* was performed using the EpiTyper by MassArray (Sequenom, San Diego, CA, USA) based upon MassCLEAVE base-specific cleavage and MALDI-TOF mass spectrometry, as recommended by the manufacturer.

### Cell Cultures

Human placental choriocarcinoma cell lines JEG-3 and human embryonic kidney cells (HEK293) were obtained from Cell Resource Center of Shanghai Institutes for Biological Sciences (Chinese Academy of Sciences, Shanghai, China). Both cell lines were repeatedly subcultured in Dulbecco’s modified Eagle’s medium (DMEM) (Gibco-BRL, Life Technology, Carlsbad, CA) supplemented with 10% fetal bovine serum and antibiotics (100 unites/ml penicillin, 100 µg/ml streptomycin) in 5% CO_2_ humidified atmosphere at 37°C.

### Constructions of Luciferase Vectors

DNA fragment encoding the human *LEP* promoter corresponding to the region from −279 to +84 was amplified through PCR. After digested with *Bgl*II/*Hind*III, the amplified fragment was purified and cloned into the promoter-less luciferase-reporter vector pGL3-Basic (Promega). A CEBPα expressing plasmid was constructed by ligation of the *CEBPα* full-length cDNA and the pcDNA3.1/myc-His(-)A expression vector (Life technologies). The primers used for DNA fragment amplifications above were summarized in Table S1.

### Plasmids CpG Methylation

10 µg of *LEP*- pGL3 plasmids were *in vitro* methylated using 40 units of the CpG DNA methylase M.SssI (New England Biolabs) incubated for 4 hours. The reaction was terminated by heating (65°C, 20 min). Methylated plasmids were purified by the DNA Clean & Concentrator Kit (Zymo Reseach). The efficiency of the methylation was examined by a *Bgl*II/*Hind*III digestion of the methylated *LEP*- pGL3 plasmids. The completeness of methylation was proven by the inhibition of *Bgl*II/*Hind*III digestion.

### Transient Transfection of Cells and Luciferase Reporter Assay

24 hours prior to transfection, JEG-3 and HEK293 cells were seeded into 24-well plates containing media without antibiotics at a concentration sufficient to give 80% confluence. Transient transfection was carried out using Lipofectamine 2000 (Life Technologies), and pRL-CMV vector was used to correct for transfection efficiency. For each well, 0.8 µg of *LEP*-pGL3 or *LEP* (methy)-pGL3 basic construct, 40 ng of pRL-CMV vector, and an optional 0.8 µg of CEBPα expression vector were diluted in 50 µl of Opti-MEM medium (Life Technologies); and 0.8 µl of Lipofectamine 2000 were diluted in 50 µl of Opti-MEM medium and incubated for 5 minutes at room temperature. After the combination of the diluted DNA and Lipofectamine 2000, the complexes were incubated for 20 min at room temperature to allow complex formation. The culture medium was replaced by 400 µl of Opti-MEM, and the plasmid-Lipo2000-OptiMEM complexes were added to the cells. Following 5 h of incubation at 37°C, 500 µl of medium containing 10% (v/v) fetal bovine serum were added to replace previous medium. Twenty-four hours after transfection, the cells were washed once with PBS and lysed for subsequent measurement of both firefly and *Renilla* luciferase activities using Dual-Luciferase Reporter Assay System (Promega) with Luminoskan TL Plus (Thermo Labsystem) according to the manufacturer’s procedures. The luciferase assay was repeated in triplicate independent experiments to reduce the variability and increase the accuracy.

### Statistical Analysis

Descriptive statistics are presented as mean values ± SD. Statistical analysis is performed using the software SPSS version 16.0 software (SPSS Inc., Chicago, IL, USA). Differences of relative gene expression between preeclamptic and normal placentas and the analysis of relative luciferase activity between groups were tested by student’s *t*-test. DNA methylation data is by excellence beta-distributed, and therefore the analysis comparing the DNA methylation in preeclamptic placentas with that of in control specimens were assessed by the non-parametric Mann-Whitney *U*-test. Gestational age was used as covariate to adjust the analysis of DNA methylation differences in PE placentas compared with controls, since these clinical characteristics significantly differed between the two groups. *p* value of less than 0.05 was considered as statistically significant.

## Results

### Overview of Gene Expression Microarray

**Figure 1:**
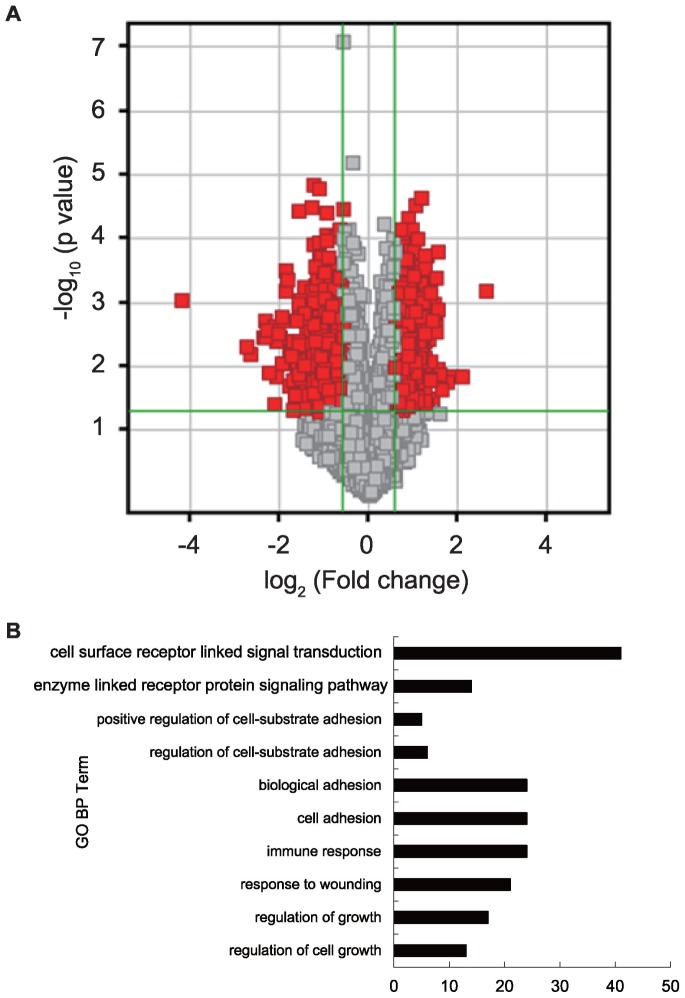
Bioinformatic analysis of the gene expression microarray results. (A) Volcano plots of genes with differential expression in pathological versus normal placentas. The *x* axis represents the log_2_ of the fold change, and the *y* axis represents the -log10 of the *p* value from a student’s *t*-test. So the red points in the plot represent the differentially expressed genes with statistical significance (with a fold change > = 1.5, and *p* value <0.05). (B) Functional annotation analysis of genes that were significantly differentially expressed between preeclamptic and normal placentas. GO, Gene Ontology; BP, Biological Process.

To search for the differentially expressed genes between pathological placentas and controls, we conducted a NimbleGen gene expression microarray analysis. Applying Student *t*-test (*p*<0.05) and a fold change criterion (>1.5 fold change in gene expression in pathological and control placentas) produced a set of 1312 genes with differential expression (Figure 1a). Among them, 387 probes (including 251 genes, 137 genes with up-regulation and 114 genes with down-regulation in preecclamptic placentas) had fold change differences greater than 2. Altogether, these genes included showed significant enrichment based on the gene ontology analysis (Figure 1b) such as regulation of cell growth, immune system and cell adhesion.

Consistent with our expectations, among the differentially expressed genes, there is a remarkable concordance of genes between our findings and other reported studies although we used different clinical samples and platforms, such as *FLT1* [5], [31], *JAG1* [32] and *ENG* [33]. We have listed the overlapped genes of our microarray data in supplementary material Table S2.

### Validation of the Differentially Expressed Genes: *LEP* and *SH3PXD2A*

**Figure 2:**
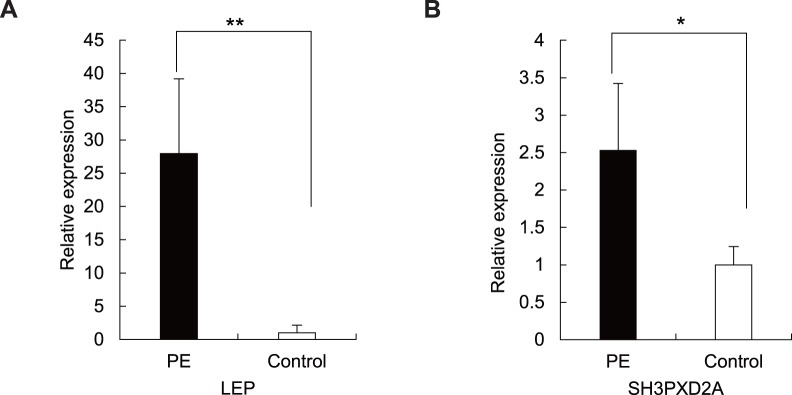
Validation the mRNA expression of *LEP* and *SH3PXD2A* in preeclamptic (n = 7) versus normal (n = 6) placentas. (A) Expression of *LEP* mRNA measured by qRT-PCR. The difference between preeclamptic placentas and normal controls is highly significant (*p* = 0.003). **p<0.01. Each bar represents the average relative expression compared with *GAPDH*. The average mRNA level of *LEP* and *SH3PXD2A* in healthy controls was defined as 1. (B) Expression of *SH3PXD2A* mRNA quantified by qRT-PCR. The expression of *SH3PXD2A* is significantly elevated in placentas from pregnancies with PE (*p* = 0.024). *p<0.05. Each bar represents the average relative expression compared with *GAPDH*. The average mRNA level of *SH3PXD2A* in healthy controls was defined as 1.

To validate the results by NimbleGen gene expression microarray, qRT-PCR was carried out in additional 7 placentas from pregnancies with PE and in 6 placentas from healthy pregnancies. We chose the gene with the greatest expression difference (*LEP*, with the fold change = 18.832) and the gene with the most significant *p* value (*SH3PXD2A*, with *p* value = 1.41×10^−5^). The results here showed that the expression of LEP was significantly elevated in preeclamptic placentas, with 27.94-fold increase, compared with that in normal placentas (Figure 2a, *p* = 0.003). As for *SH3PXD2A*, it is consistent with our expectation that the expression increased in placentas from pregnancies with PE 2.53-fold compared with that in normal placentas (Figure 2b, *p* = 0.024).

### DNA Methylation Analysis of *LEP* and *SH3PXD2A*

It is quite clear that DNA methylation has been a potentially important mechanism to regulate gene expression. In order to explore whether the different expressions of *LEP* and *SH3PXD2A* were influenced by the effect of DNA methylation, and to have a better understanding of the mechanism underlying the occurrence of PE, we made further investigation of DNA methylation of the relevant genes.

**Figure 3:**
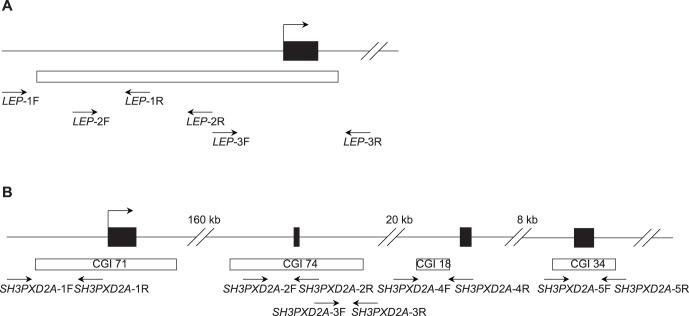
Schematic representation of *LEP* and *SH3PXD2A* gene. (A) Maps of the TSS and the proximal promoter region of the human *LEP* gene. Positions and orientation of the MassARRAY primers are indicated by black arrows. (B) Maps of *SH3PXD2A* locus presenting the CGIs region in the promoter region (CGI71) and in the gene body (CGI74, CGI18 and CGI34). Positions and orientation of the MassARRAY primers are indicated by black arrows.

We analyzed the methylation patterns of *LEP* and *SH3PXD2A* in 32 placentas (16 pathological versus 16 normal placentas) using the high-throughput MALDI-TOF MS assay (Sequenom). The gene maps of *LEP* locus and *SH3PXD2A* locus were shown in Figure 3. The analyzed amplicons in the study comprised the CpG island (CGI) region in both genes. For *LEP*, three amplicons were designed to cover the whole CGI region, in which 62 CpG sites (40 units) per sample were amenable to be analyzed (Figure 3a). For *SH3PXD2A* which contains 6 CGIs, its upstream 4 CGIs (CGI71, CGI74, CGI18 and CGI34) were analyzed in the study and 88 CpG sites (54 units) per sample were able to be detected (Figure 3b).

**Figure 4:**
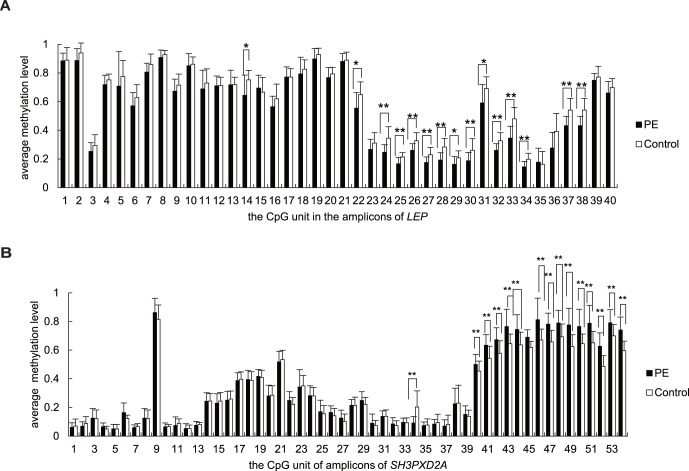
Quantitative DNA methylation of *LEP* and *SH3PXD2A* in all PE (n = 16) and control (n = 16) samples. (A) The average methylation level of the CGI of *LEP*. Several CpG sites in the vicinity of TSS (unit 34) are all significantly hypomethylated in the preeclamptic placentas. Position with significantly differential methylation between PE and control groups is marked by asterisks. **p<0.01, *p<0.05. (B) The average methylation level of CGIs of *SH3PXD2A*. The TSS CGI comprised the CpG sites from unit 1 to13; The CGI74 comprised the CpG sites from unit 14 to 30; The CGI18 comprised the CpG sites from unit 31 to 39; The CGI34 comprised the CpG sites from unit 40 to 54. Position with significantly differential methylation between PE and control groups is marked by asterisks. **p<0.01, *p<0.05.

In the *LEP* gene, most of the CpG sites in amplicon 1 and 2 are at a high degree of methylation (average methylation level >0.6 except unit 3), while the CpG sites in amplicon 3 around TSS show a low degree of methylation. Interestingly, we found that CpG dinucleotides situated around TSS [such as CpG sites determined to bind Sp1 (unit 28, average methylation = 0.193, 0.284 in preeclamptic vs normal placentas respectively, *p* = 1.57×10^−4^), LP1 (unit 29, average methylation = 0.163, 0.208 in preeclamptic vs normal placentas respectively, *p* = 0.023) and CEBPα (unit 31, average methylation = 0.591, 0.689 in preeclamptic vs normal placentas respectively, *p* = 0.031) and CpG sites in the position of TSS (unit 34, average methylation = 0.145, 0.198 in preeclamptic vs normal placentas respectively, *p* = 0.001)] were significantly hypomethyalted in the placentas from pregnancies with PE (Figure 4a). In addition, we further tested the correlation between the DNA methylation and gene expression data, the result showed that LEP expression and differential *LEP* methylation was negatively correlated, but did not reach significant level (Figure S1).

In the *SH3PXD2A* gene, the CpG sites of the promoter region (amplicon 1) in both preeclamptic placentas and normal placentas are almost unmethylated with no statistical difference, which is in line with the hypothesis that the promoter CGI generally remains unmethylated. The methylation patterns in the gene body of *SH3PXD2A* (from unit 14 to 54) were shown in Figure 4b. All the CpG sites in CGI74 region (from unit 14 to 30) were also at a low methylation with no statistical difference between preeclamptic and control placentas. One CpG site (unit 34) in CGI 18 (from unit 31 to 39) were significantly hypomethylaed in pathological placentas than that in normal placentas (the average methylation = 0.093, 0.203 in PE and control respectively, *p* = 0.002). The remaining CpG sites in the CGI18 were also at a low methylation level. Interestingly, the CpG sites in the CGI34 region (Figure 4b, from unit 40 to 54) as a whole were at a high methylation level (the mean methylation = 0.725, 0.616 in PE and control respectively, *p* = 1.57×10^−4^), most CpG sites are higher methylated in preeclamptic placentas than those in controls except unit 40 (including CpG 1 and CpG2), of which, unit 41, 42, 43, 44, 45, 47, 48, 49, 50, 51, 52, 53 and 54 were statistically significant (*p* = 0.003, 0.006, 0.007, 0.003, 1.90×10^−4^, 1.29×10^−4^, 0.004, 0.002, 0.007, 0.002, 1.29×10^−4^, 0.003 and 4.62×10^−5^ respectively, Figure 4b). The correlation between DNA methylation level in CGI34 region and gene expression of *SH3PXD2A* in normal placentas was also performed. The result showed that gene body methylation and gene expression of *SH3PXD2A* was positively correlated without significance (Figure S2).

Clinical characteristic gestational age presented significant differences between groups, which might have influence on the statistical result. To adjust this, covariance analysis was performed. Our results indicated that the statistical significance of most CpG units showed no obvious difference after the correction except for unit 2 and unit 36 in *LEP* gene (Table S3).

### Luciferase Analysis of DNA Methylation and Transcriptional Activity of *LEP*

**Figure 5:**
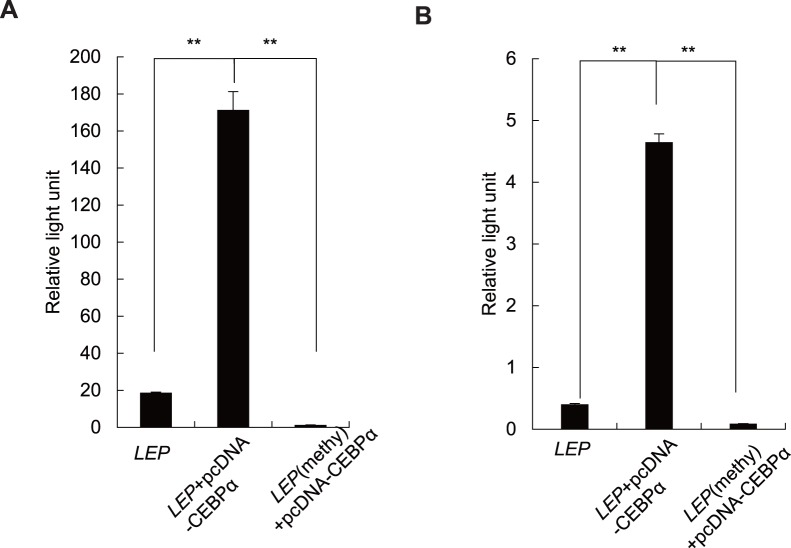
Luciferase reporter analysis of the transfection in cell lines. (A) Luciferase activity of *LEP*-pGL3 and *in vitro* methylated *LEP* (methy)-pGL3 constructs in JEG-3 cells; (B) Luciferase activity of *LEP*-pGL3 and in vitro methylated *LEP* (methy)-pGL3 constructs in HEK293 cells.

To identify the effect of DNA methylation on the transcriptional activity of *LEP* promoter in placenta, the region (−279_+84bp) placed 5′ adjacent to the reporter luciferase gene was in vitro methylated to further analyze its transcriptional activity in both JEG-3 and HEK293 cells. Methylation of CpG site within the CEBPα binding site participated in the down-regulation of LEP expression in adipose cells [34]. Hence, in our study, we cotransfected the cells with the target plasmids *LEP*-pGL3 or *LEP* (methy)-pGL3 and an expression vector pcDNA-CEBPα. Consistent with our expectation, the relative light unit of CpG DNA methylase M.SssI methylated construct showed a striking decrease compared with that in untreated construct in both cell lines (the relative light unit is 1.04, 171.16 in JEG-3 cells respectively, *p* = 8.03×10^−6^, Figure 5a; the relative light unit is 0.08, 4.64 in HEK293 cells respectively, *p* = 6.21×10^−7^, Figure 5b).

In addition, the relative light unit of transfection containing expression vector pcDNA-CEBPα is significantly stronger than that with *LEP*-pGL3 construct only (the relative light unit is 171.16 and 18.46 respectively, *p* = 1.24×10^−5^, Figure 5a; the relative light unit is 4.64, 0.39 in HEK293 cells respectively, *p* = 1.34×10^−5^, Figure 5b), which indicated that the transcription factor CEBPα is also necessary for the transcription of *LEP* in placental cells.

## Discussion

In the present study, we applied gene expression microarray analysis to PE and control groups to search the candidate genes for following DNA methylation analysis. To control the maternal age and body mass index of pregnancies, the sample size used in the expression profile is small (5 placentas with PE and 7 controls), which might contribute to so many differentially expressed genes. However, among the differentially expressed genes, our current study and previous microarray analysis share a series of common genes listed in supplementary material Table S2. The homogeneity identified in our study suggested the effectiveness of our microarray analysis and the value to take further research. Based on the bioinformatics analysis, significantly enriched gene sets and pathways, including cell adhesion, immune response and pathways in cancer were identified through gene ontology (GO) database (Figure 1b) and KEGG database. They functionally suggested that the disease was heterogeneous and multi-factorial, that previous mentioned pathogenic theories such as the immune maladaptation between the mother and the fetus [7], [8] and the impaired invasion of the extravillous trophoblast [35] are contributive to the occurrence of PE.

It is widely accepted that promoter DNA methylation plays a role in the regulation of gene expression in mammalian cells and is considered as an important contributor to disease states [36]. Among the genes with expression difference, *LEP* showed significant differences in DNA methylation in the CpG sites in the vicinity of TSS including the binding sites of transcription factors Sp1, LP1 and CEBPα in preeclamptic placentas compared with that in controls. Previous study in adipose cells [34] and our luciferase reporter assay in JEG-3 cells both pointed out that CEBPα expression was needed for stimulation of *LEP* promoter transcriptional activity and the methylation patterns of the CpG in the binding site of CEBPα participated in the regulation of *LEP* expression. As a result, we speculated that the demethylation in the corresponding CpG sites might influence the binding of the transcription factors, thereby led to the altered LEP expression in placentas from pregnancies with PE.

Leptin, the encoding protein of the *LEP* gene [37], which possesses pleiotropic effects with regulatory function in reproductive maturity and fertility such as regulation of ovarian function and implantation by promoting proliferation and survival of trophoblastic cells [38], has been repeatedly proven to be up-regulated not only in preeclamptic placentas [39]–[43], but also in the maternal plasma of pregnancies with PE [44]. In this regard, leptin must have played an important role in the development of PE, and it is necessary to reiterate the need for further research dedicated to elucidating its role in PE. Our study is so far the first report about the significant relationship between *LEP* proximal promoter methylation and PE. The small correlation coefficient between the DNA methylation and gene expression data is partially due to the small sample size used in the correlation study, which may also suggest that other mechanisms besides DNA methylation may involve in the regulation of gene expression. Previous studies have demonstrated that the hormone hCG [45], estrogens [46], cAMP [47] and glucocorticoid response elements [48] are also confirmed to be responsible for the regulation of leptin expression. As a result, we have predicted that DNA methylation was a potential approach in the regulation of *LEP* expression.

*SH3PXD2A*, also known as *FISH*, *TKS5* or *SH3MD1*, located on 10q24.33, is predicted with several transcriptive isoforms. Researches have stated that the distribution of the CGI is obligatory for understanding the functions of DNA methylation [49]. Consequently, current study measured the methylation level of 3 CGIs (CGI74, CGI18 and CGI34) of *SH3PXD2A* in the gene body besides the one (CGI71) in the proximal promoter region. Interestingly, our results presented that nearly all the CpG sites were significantly hypermethylated in the preeclamptic placentas with high methylation (methylation level in all CpG sites >0.6) in CGI34 region. Prior research has pointed out that the methylation in gene body CGI, unlike the CGI in TSS, is positively correlated with gene expression [50]. Hence, the hypermethylated CGI34 perhaps participated in the upregulation of SH3PXD2A expression in placentas from pregnancies with PE.

The encoding product of *SH3PXD2A* is a scaffolding protein and Src kinase substrates comprised of an amino-terminal Phox homology (PX) domain and five Src homology 3 (SH3) domains, as well as multiple motifs for binding both SH2 and SH3 domaincontaining proteins [51]. As yet, there are no reports concerning SH3PXD2A for PE. Of all the published data about *SH3PXD2A*, it is worthy to note the role of the encoding protein by *SH3PXD2A* in invadopodia or podosome formation that promotes cell metastasis [52]–[54]. Placenta is of pseudo-malignant nature, and trophoblast invasion is important for its formation. Patel *et al.* have reported the extravillous trophoblasts regulated extracellular matrix degradation through formation of atypical podosomes [55], which are of protrusive structures formed on the trophoblasts, and are also actin-rich and are able to degrade the extracelluar matrix during the invasion. The degradation ability and dynamics of the structures have the characteristics of both podosome and invadopodia. The product encoding by *SH3PXD2A* may participated in the invasion of trophoblast cells in the formation of placenta, and PE is hallmarked by the deregulation of the trophoblast invasion, in this regard, we therefore speculated that the encoding protein by SH3PXD2A might take part in the development of PE.

It is worthy to note that the placentas used in our present study were all from pregnancies after delivery. Consequently, the differentially expressed genes in the microarray analysis were either responsible for the origin of PE or the consequence of the disease after the onset of PE. In summary, we adopted the gene expression microarray analysis between groups to search for the candidate genes for following DNA methylation analysis. The aberrant methylation of the interesting gene candidates *LEP* and *SH3PXD2A* might partially contribute to the deregulation of their expression. We are the first to report the relationship between *LEP* proximal promoter methylation and PE and our results firstly proposed an unexpected link between *SH3PXD2A* and the development of PE. The more detailed role of *LEP*, and especially *SH3PXD2A* need to be further studied in our following work.

## Supporting Information

Figure S1 **Correlation of differential** ***LEP*** **DNA methylation with gene expression.** To increase the sample size of the correlation analysis and reduced the possible effect of the disease status, the linear correlation was performed based on normal placentas used in microarray and q-PCR analysis.
(EPS)

Figure S2 **Correlation of differential** ***SH3PXD2A*** **DNA methylation with gene expression.** To increase the sample size of the correlation analysis and reduced the possible effect of the disease status, the linear correlation was performed based on normal placentas used in microarray and q-PCR analysis.
(EPS)

Table S1 **Sequences of PCR primers used in this study.**
(DOC)

Table S2 **The overlapping genes in our microarray analysis with other published microarray papers.**
(DOC)

Table S3 **Summary of adjusted and unadjusted statistical analyses for the CpG units of** ***LEP*** **and** ***SH3PXD2A*** **genes by gestational age.**
(DOC)
