# Supplementary material for: Up-Regulated Expression and Aberrant DNA Methylation of LEP and SH3PXD2A in Pre-Eclampsia
Source: PLoS One. 2013 Mar 27;8(3):e59753. doi: 10.1371/journal.pone.0059753 (PMC3609796; doi:10.1371/journal.pone.0059753)
Supplement: Table S1 — Sequences of PCR primers used in this study. (DOC) [file pone.0059753.s003.doc]

**Table S1 Sequences of PCR primers used in this study**

| **Gene** | **Primer*** | **Sequence(5'-3')** | **Product Size (bp)** |
| --- | --- | --- | --- |
| **MassARRAY** |  |  |  |
| *LEP-1* | tag-FW | tag-GGAGTTGAGGATGGAGATTTATAGT | 244 |
|  | T7-RV | T7-CCAAAAAAACCCAAAATTAAAAAAA |  |
| *LEP-2* | tag-FW | tag-AGATAATTTGTTTTGAGGTTTGGAA | 356 |
|  | T7-RV | T7-AAAATCCTTAATATCCCTCCAAAAA |  |
| *LEP-3* | tag-FW | tag-TAAATTTTTGGGAGGTATTTAAGGG | 471 |
|  | T7-RV | T7-ACACAACCCAACAACAAATCC |  |
| *SH3PXD2A-1* | tag-FW | tag-GGATATAATGGTTTTATGAGGGAAATTA | 495 |
|  | T7-RV | T7-CCTTACCCCTCCCAACTATAAAAT |  |
| *SH3PXD2A-2* | tag-FW | tag-TTTAAGAGGAAATTAGGTAAGGGAGTT | 282 |
|  | T7-RV | T7-CCCACACCCACTAAAAACAAC |  |
| *SH3PXD2A-3* | tag-FW | tag-TTTTTTGGAATTGTTATAGTTTTTG | 355 |
|  | T7-RV | T7-CAATTTAAAAAATAAAAAAAACCTACCTC |  |
| *SH3PXD2A-4* | tag-FW | tag-GGAGGAGGGAGGAATAAAAATTAGT | 447 |
|  | T7-RV | T7-ACCCCCTAACTCCCCATATC |  |
| *SH3PXD2A-5* | tag-FW | tag-TAGAAAGGAAGGGTTAGGTTGTTTT | 288 |
|  | T7-RV | T7-AAACAAACAAAAAATCCACAATAAA |  |
| **qPCR** |  |  |  |
| *LEP* | FW | TGCCTTCCAGAAACGTGATCC | 164 |
|  | RV | CTCTGTGGAGTAGCCTGAAGC |  |
| *SH3PXD2A* | FW | GGACCCCAAGCAAAGGATCAT | 115 |
|  | RV | TGCCCGGCAGTATTCATCG |  |
| *GAPDH* | FW | AAGGTGAAGGTCGGAGTCAAC | 102 |
|  | RV | GGGGTCATTGATGGCAACAATA |  |
| **Vector Construct** |  |  |  |
| *LEP*-pGL3 | FW | GGAAGATCTGAGCCTCTGGAGGGACATCAAGGAT | 381 |
|  | RV | CCCAAGCTTTGGCCTGCCAAGAAAGACCAG |  |
| *CEBPα* | FW | CCGGAATTC ATGGAGTCGGCCGACTTCTA | 1095 |
|  | RV | CCCAAGCTTCGCGCAGTTGCCCATGGC |  |

^a^ FW, forward; RV, reverse.

tag, aggaagagag

T7, cagtaatacgactcactatagggagaaggct
