# Supplementary material for: Up-Regulated Expression and Aberrant DNA Methylation of LEP and SH3PXD2A in Pre-Eclampsia
Source: PLoS One. 2013 Mar 27;8(3):e59753. doi: 10.1371/journal.pone.0059753 (PMC3609796; doi:10.1371/journal.pone.0059753)
Supplement: Table S2 — The overlapping genes in our microarray analysis with other published microarray papers. (DOC) [file pone.0059753.s004.doc]

**Table S2 The overlapping genes in our microarray analysis with other published microarray papers**

| Gene Symbol | RefSeq | Fold Change direction | PubMed IDs^a^ |
| --- | --- | --- | --- |
| *BCL6* | NM_001706 | ↑ | 18533121,19249095,21810232 |
| *CCK* | NM_000729 | ↑ | 19027158 |
| *CDO1* | NM_001801 | ↑ | 18533121 |
| *CRH* | BC002599 | ↑ | 18818296, 21079238 |
| *EBI3* | NM_005755 | ↑ | 18533121, 21430563 |
| *ENG* | NM_000118 | ↑ | 18330824,18818296,19249095, 21183218, 21810232 |
| *FLRG* | NM_005860 | ↑ | 21810232 |
| *FLT1* | NM_002017 | ↑ | 17616861,18330824,18533121,18818296,19249095,19787364,21183218, 21430563,21810232 |
| *FLT4* | NM_182925.4 | ↑ | 19249095 |
| *FSTL3^b^* | NM_005860 | ↑ | 19027158 |
| *HCG* | [NM_033043](http://genome.ucsc.edu/cgi-bin/hgTracks?position=chr19:49547102-49548568&hgsid=318241031&refGene=pack&hgFind.matches=NM_033043,) | ↑ | 19249095,22472943 |
| *HTRA1* | NM_002775 | ↑ | 21430563,18818296 |
| *INHA* | NM_002191 | ↑ | 18533121,18818296,19249095,21183218,21810232 |
| *INHBA* | NM_002192 | ↑ | 20204130,21810232 |
| *INSL4* | NM_002195 | ↑ | 18533121 |
| *LEP* | NM_000230.2 | ↑ | 18533121,18818296,19249095,19787364,20204130,20541258,21183218,21430563,21810232 |
| *PAPPA2* | NM_020318 | ↑ | 18818296, 19249095，20541258，21183218， 21810232 |
| *RDH13* | NM_138412 | ↑ | 21183218 |
| *SIGLEC6* | NM_001245 | ↑ | 18818296，20541258，21183218，21430563 |
| *TREM1* | NM_018643 | ↑ | 20541258 |
| *ANPEP* | BC058928 | ↓ | 17616861 |
| *CX3CR1* | NM_001337 | ↓ | 22472943 |
| *FOSB* | NM_006732 | ↓ | 22472943 |
| *JAG1* | NM_000214 | ↓ | 17616861 |
| *VCAM1* | NM_001078 | ↓ | 22472943 |

Fold change direction ↑ referred to as the gene with upregulation in the placentas from pregnancies with PE;

Fold change direction ↓ referred to as the gene with downregulation in the placentas from pregnancies with PE.

^a^ The published paper summarized in this study all referred to that mentioned in the review article by Louwen et al [26].

^b^ The gene was downregulated in preeclamptic placentas in the study mentioned in the review article by Louwen et al [26], which is upregulated in our present microarray analysis.
