# Supplementary material for: Up-Regulated Expression and Aberrant DNA Methylation of LEP and SH3PXD2A in Pre-Eclampsia
Source: PLoS One. 2013 Mar 27;8(3):e59753. doi: 10.1371/journal.pone.0059753 (PMC3609796; doi:10.1371/journal.pone.0059753)
Supplement: Table S3 — Summary of adjusted and unadjusted statistical analyses for the CpG units of LEP and SH3PXD2A genes by gestational age. (DOC) [file pone.0059753.s005.doc]

**Table S3 Summary of adjusted and unadjusted statistical analyses for the CpG units of *LEP* and *SH3PXD2A*** genes by gestational age

| *LEP* | | | *SH3PXD2A* | | |
| --- | --- | --- | --- | --- | --- |
| CpG position | Unadjusted *p*-value | Adjusted *p*-value | CpG position | Unadjusted *p*-value | Adjusted *p*-value |
| unit 2 | 0.037 | 0.13 | unit 34 | 0.002 | 0.006 |
| unit 14 | 0.019 | 0.003 | unit 41 | 0.003 | 0.006 |
| unit 22 | 0.012 | 0.039 | unit 42 | 0.006 | 0.001 |
| unit 24 | 0.001 | 1.4×10^-4^ | unit 43 | 0.007 | 0.001 |
| unit 25 | 0.003 | 0.004 | unit 44 | 0.003 | 0.004 |
| unit 26 | 0.002 | 0.002 | unit 45 | 0.00019 | 4.4×10^-3^ |
| unit 27 | 0.005 | 0.003 | unit 47 | 0.00013 | 2.9 ×10^-3^ |
| unit 28 | 1.6×10^-3^ | 2.2×10^-4^ | unit 48 | 0.004 | 0.003 |
| unit 29 | 0.023 | 0.01 | unit 49 | 0.002 | 0.003 |
| unit 30 | 0.01 | 0.002 | unit 50 | 0.007 | 0.001 |
| unit 31 | 0.031 | 0.046 | unit 51 | 0.01 | 0.013 |
| unit 32 | 0.002 | 0.002 | unit 52 | 0.00013 | 3.2 ×10^-3^ |
| unit 33 | 4.6×10^-4^ | 2.0×10^-3^ | unit 53 | 0.003 | 0.007 |
| unit 34 | 0.001 | 0.001 | unit 54 | 0.000046 | 5.8×10^-4^ |
| unit 36 | 0.019 | 0.083 |  |  |  |
| unit 37 | 3.3×10^-3^ | 3.6×10^-3^ |  |  |  |
| unit 38 | 3.3×10^-3^ | 3.6×10^-3^ |  |  |  |
